# ‘They're getting a taste of our world’: A qualitative study of people with multiple sclerosis' experiences of accessing health care during the COVID‐19 pandemic in the Australian Capital Territory

**DOI:** 10.1111/hex.13284

**Published:** 2021-07-06

**Authors:** Anne Parkinson, Janet Drew, Sally Hall Dykgraaf, Vanessa Fanning, Katrina Chisholm, Mark Elisha, Christian Lueck, Christine Phillips, Jane Desborough

**Affiliations:** ^1^ Department of Health Services, Research and Policy Australian National University Canberra ACT Australia; ^2^ Medical School Australian National University Canberra ACT Australia; ^3^ Department of Neurology Canberra Hospital Canberra ACT Australia

**Keywords:** multiple sclerosis, pandemic, patient experience, qualitative, risk assessment, telehealth

## Abstract

**Background:**

People with multiple sclerosis (MS), who are often immunocompromised, require complex care and engage with a variety of health‐care providers to manage their health.

**Objective:**

To elucidate people with MS' experiences of accessing health care during the COVID‐19 pandemic in Australia.

**Design:**

A qualitative study involving semi‐structured interviews and thematic analysis.

**Settings and participants:**

Eight adults with a clinical diagnosis of MS participated in telephone or video call interviews between June and July 2020.

**Results:**

Participants were aware that having MS made them more vulnerable to contracting COVID‐19. In some cases, usual care was postponed or not sought. Some circumstances warranted the risk of a face‐to‐face consultation. Benefits of telehealth consultations included improved access, convenience and being contact‐free. In comparison with video consultations, those via telephone were considered less personal and limited capacity to read body language, and for physical examination. Most participants hoped to incorporate telehealth into their future health‐care routines.

**Discussion and conclusion:**

Personal risk assessment and trust in health‐care professionals are determinants of the mode through which people with MS accessed health care during the COVID‐19 pandemic. Telehealth has been a valuable tool to mitigate COVID‐19 transmission through enabling contact‐free consultations. People with MS may find specific value in video consultations, which enable visualization of physical function. There is a need for training and support for all clinicians to conduct remote consultations.

**Patient or public contribution:**

This study was conducted by a team comprised of four people with MS, a neurologist and four health services researchers.

## 1 BACKGROUND

The emergence of COVID‐19, and subsequent declaration of a pandemic in March 2020 by the World Health Organization, has greatly impacted health services globally.^1^ Neglect or postponement of ‘usual care’ can be an unintended consequence of prioritizing emergency responses during epidemics, pandemics and natural disasters, leading to increased all‐cause morbidity and mortality.^2^, ^3^, ^4^ People with chronic conditions benefit from continuity of routine care and are at increased risk of death or complications if their treatment is interrupted.^5^, ^6^, ^7^ One of the greatest challenges facing health services during the pandemic is an invisible one, specifically how best to provide care for those who might fear seeking it.^8^

Australia's health system response to COVID‐19 aimed to protect vulnerable populations by supporting the continued provision of usual care to the whole community for all health conditions.^9^ The response included the rapid implementation of telehealth via telephone or video, publicly funded through Australia's universal public health‐care scheme, Medicare and the related Medical Benefits Schedule (MBS). This enabled access to both COVID‐19‐related and regular health care, at the same time protecting vulnerable patients and clinicians, and minimizing COVID‐19 transmission in health‐care settings.^10^ MBS items were expanded to cover telehealth consultations with general practitioners (GP), some nurses and midwives, allied health‐care providers (AHCP) and specialist medical consultants.^10^, ^11^

Multiple sclerosis (MS) is a chronic, inflammatory neurological condition characterized by a range of symptoms which can lead to permanent disability.^12^ It is also the most common inflammatory neurological disease of young people.^13^ People with MS constitute a vulnerable population.^10^ In Australia, there are over 25 000 people with MS and its prevalence is increasing as are the associated costs.^14^ People with MS are high users of health‐care services and often engage with a broad range of health‐care providers as part of managing their physical and mental health.^15^ Subsequently, ease of access to health‐care services and affordability are regarded as a high priority.^16^ The COVID‐19 pandemic has affected access to health‐care providers for people with MS: a recent UK survey of MS health professionals, including nurses, physiotherapists and occupational therapists found that the health‐care needs of people with MS were not being met during the pandemic, particularly with respect to accessing rehabilitation services.^17^ In Italy, a survey of 2722 people with MS reported disruption to usual health‐care and social services, negatively impacting their overall health and wellbeing.^18^ And, in the United States, a survey of over 1000 people with MS found many had cancelled or postponed medical visits, including visits to neurologists due to concerns about possible infection.^19^ Further compounding concern, many people with MS are on disease‐modifying therapies (DMTs), which may increase their risk of infection or serious complications due to COVID‐19.^19^ While on‐going access to health care is essential for the management of MS, safe access was a priority for people with MS during the COVID‐19 pandemic.

Our aim was to examine the experiences of people with MS in accessing health care, including via telehealth, during the COVID‐19 pandemic in Australia following the Australian government's expansion of subsidized telehealth consultations.

## 2 METHODS

A pragmatic, qualitative descriptive approach, often employed in health services research,^20^ was used to optimize participants' experience, using language similar to their own. This methodology uses interview guides based on expert knowledge, which in our study was informed by experts in living with MS. ‘Staying close to the data’,^20^ we used thematic analysis in accordance with the six steps described by Braun and Clarke,^21^ including data familiarization, generation of codes, collation of themes, thematic review, definition of themes and reporting.^21^ Two researchers (AP and JDr) familiarized themselves with the data: listening to the recordings and reading the transcripts multiple times. This grounding in the data informed identification of codes and subsequent generation of themes after which review and definition of themes were undertaken in collaboration with two other team members (SH and JD).

Following the principles of patient and public involvement (PPI) in research,^22^, ^23^ our research team, which we refer to as the Health Experience Team (HET), was formed to embed the perspectives of people living with MS in all aspects of the Australian National University's Our Health in Our Hands (OHIOH) personalized medicine programme.^24^ The HET is a multidisciplinary team with expertise in both qualitative and quantitative methodologies and comprises a neurologist, health services researchers and people living with MS. Four members of the HET who have MS contributed to the study design, including development of the interview protocol, analysis and writing the paper. Interviews were conducted by a member of the HET with MS (JDr) who is a MS peer support volunteer, has undertaken training to provide peer support and is completing a Master of Philosophy (Population Health). Her preparation included auditing a qualitative research master's‐level course, following which she discussed interview techniques with an experienced researcher (AP). They jointly conducted the first interview and debriefed afterwards discussing and reflecting on the experience. She conducted subsequent interviews alone.

There are great similarities between PPI and participatory action research (PAR), both of which question the nature of knowledge and affirm that experience is an important and legitimate form of knowledge, and share the philosophy of valuing collaboration and partnership^25^; however, the latter emphasizes a more hands‐on approach in terms of actively involving community participants in study design and the collection and analysis of data.^26^ The deep involvement of our MS research partners in this study is more reflective of PAR methodology.

This study was approved by the Australian National University Human Research Ethics Committee (2020/237).

### 2.1 Recruitment and participants

We recruited a convenience sample of eight people living in the Australian Capital Territory (ACT), with a clinical diagnosis of MS, from a pool of people who had either participated in previous research with our group and/or had previously registered an expression of interest for involvement in research about MS as part of the OHIOH programme^24^ (n = 49). Participants were purposively chosen in an attempt to include a gender and age balance and to include people who had not previously participated in studies with our group. In order to pace data collection, invitations were sent weekly for three weeks to five or six people from our register. Invitations were sent to 17 people, nine did not reply, and eight accepted our invitation to participate. All participants were provided information about the study and gave informed written consent prior to interviews. Participants' ages ranged from 30 to 59 years. Participants had been diagnosed between three and 27 years previously, were at differing stages of MS progression and used different medications. Participants' mobility was varied and ranged from active and able to requiring mobility aids. Expanded Disability Status Scale (EDSS) scores were not available. Participant characteristics are presented in Table 1.

**Table 1:** Participant characteristics

ID no.	Age (y)	Gender	Type of MS	Year of diagnosis	Disease‐modifying therapy (DMT)	Choice of interview mode	Mobility
1	30‐39	F	RRMS	2014	Glatiramer acetate	Online video	Active and able
2	30‐39	F	RRMS	2017	Ocrelizumab	Telephone	Active and able
3	40‐49	M	RRMS	2013	Natalizumab	Online video	Active and able
4	50‐59	M	SPMS	1993	None	Online video	Requires mobility aid
5	20‐29	F	RRMS	2012	Fingolimod	Telephone	Requires mobility aid
6	50‐59	F	SPMS	2001	Glatiramer acetate	Telephone	Requires mobility aid
7	30‐39	F	RRMS	2018	Ocrelizumab	Telephone	Active and able
8	50‐59	F	RRMS	2009	None	Telephone	Active and able

Note

Requires mobility aid (chair and/or walking sticks).

Abbreviations: RRMS, relapsing‐remitting MS; SPMS, secondary progressive MS.

### 2.2 Data collection

Qualitative in‐depth semi‐structured interviews were conducted between June 2020 and July 2020 by two researchers (AP and JDr). The interviews were conducted at a time convenient for participants. Due to COVID‐19 restrictions, all interviews were contact‐free, and participants were offered either a telephone (n = 5) or video call via Zoom (n = 3). No differences in quality were noticed between telephone or video interviews when reading the transcripts. Most participants opted for telephone interviews. The main interviewer (JDr) reflected that she preferred to conduct interviews by telephone as she felt more able to concentrate on the questions and responses rather than visual elements of the interaction. Individual interviews lasted 30‐90 minutes and were audio‐recorded. Both interviewers felt that data saturation had been reached after six interviews. A further two interviews were conducted to confirm no new concepts emerged. For exemplar questions from the guide, see Table 2.

**Table 2:** Exemplar questions

Which doctors or other health‐care professionals do you usually see to help manage your health and how often you see them?
Can you tell me about any video or phone consultations you have had since mid‐March 2020? Which ones were good/not so good and why?
Have any of your regular health‐care appointments been cancelled or postponed since mid‐March when telehealth consultations were introduced? Which ones and why? How did you feel about that?
Are you worried about COVID‐19 and if so, what sorts of things are you concerned about? Have you made any changes to your life?
In addition to the social distancing measures advised by the government, have you put in place other measures to protect yourself?
Thinking about all that has happened since mid‐March 2020, do you feel your health has been managed as well as it could have been during this period? Why?
Follow‐up questions
Thinking about having a telehealth consultation: Do you think a video meeting where you can see the doctor's face is better than talking on the phone, or just the same? Why do you think this? Do you think it is any different depending on who you are seeing for example, your GP or your neurologist or another type health‐care professional? Why do you think this?

### 2.3 Data analysis

Two researchers (AP and JDr) read the interview transcripts multiple times to ensure data familiarity and then independently coded two transcripts each. This was followed by discussion to agree on a preliminary coding framework that was then applied to the remaining transcripts. On‐going discussions were held with two further team members (SH and JD) to finalize data categories, agree on any necessary changes and determine relevant themes. Themes were then reviewed, discussed and approved by all researchers. NVivo 12 software^27^ was used for coding, analysing and managing the data.

### 2.4 Rigour

Trustworthiness and credibility of the research were established in accordance with Lincoln and Guba.^28^ Team discussions held during data analysis, including review of transcripts and identification of themes, ensured dependability. Credibility of the data was established through ascertaining saturation. This was confirmed through conducting two additional interviews and we directly linked participant's quotes with our interpretation of the data to corroborate our analysis. Authenticity was established through verbatim transcription and confirmation of this through repeated listening to recordings. We have also acknowledged potential limitations to the transferability of our findings.

## 3 RESULTS

All participants had attended consultations with a range of health‐care professionals since the pandemic was declared in March 2020, and across multiple modalities including face‐to‐face, telephone and video (see Table 3). While all participants had consulted with a general practitioner (GP), all of these consultations were by telephone rather than via video. Five participants had more than one consultation with a GP. Participants attended both video and telephone consultations with neurologists. Allied health‐care providers (AHCP) offered various options. Six participants made use of their services on a regular basis attending between twice weekly and fortnightly depending on the AHCP and their needs. Psychologists offered face‐to‐face, telephone and video consultations. Exercise physiologists transitioned to video consultations; however, one participant received home visits. Physiotherapists provided video and telephone consultations, as well as face‐to‐face where necessary including home visits for one participant. Participants ceased using massage therapists. Three participants attended hospital for treatments.

**Table 3:** Summary of consultations

Health‐care professional consulted and modality	Participant ID							
	#1	#2	#3	#4	#5	#6	#7	#8
GP face‐to‐face	✓	✓	✓	✓	✓	—	✓	✓
GP telehealth via telephone	—	—	—	—	—	✓	✓	✓
GP telehealth via video	—	—	—	—	—	—	—	—
MS nurse telehealth via telephone	—	✓		—	—	—	—	—
AHCP face‐to‐face	—	—	✓	✓	—	✓	✓	✓
AHCP home visit						✓		✓
AHCP telehealth via telephone	—	—	✓	—	✓	—	—	—
AHCP telehealth via video	—	—	—	—	✓	✓	—	—
Neurologist face‐to‐face	—	—	—	—	—	—	✓	—
Neurologist telehealth via telephone	—	—	—	—	—	✓	—	—
Neurologist telehealth via video	—	—	✓	✓	—	—	—	—
Hospital services face‐to‐face (including magnetic resonance imaging (MRI), clinic visit and initial *Hospital in the Home* consult)	—	—	—	✓	—	✓	✓	—
*Hospital in the Home* visit to patient's home; face‐to‐face	—	—	—	✓	—	—	—	—
Other specialist telehealth via video	—	—	—	—	✓	—	—	—

Abbreviations: AHCP, allied health‐care providers; GP, general practitioner (including psychologists, neuropsychologists, physiotherapists, exercise physiologists and massage therapists).

Three overarching themes emerged from the interviews: assessing personal risk, postponing usual care and new ways of accessing care.

### 3.1 Assessing personal risk

People with MS were accustomed to continual assessment of personal risk in their lives for a number of reasons; for some, this was related to on‐going immune suppression related to medication, and for others, risk was related to the potential for falls and exacerbating fatigue. They considered themselves to be experts at managing their own risk and making decisions about their health, observing that this was a new experience for the broader public during the COVID‐19 pandemic.

[W]ith this pandemic, everyone's getting a bit of a taste of what it is like to [have MS], you know, everyone's talking about health. And that's not really any different for us. They're getting a taste of our world in some respects. (#P2)

Participants had actively sought information and were aware that MS made them more vulnerable to poor outcomes if they were to contract COVID‐19, especially if they were treated with DMTs.

I'm very concerned about what would happen to me if I did contract the virus… [N]ot only would I be sick, but I'd be doubly sick because I'd get exacerbated MS symptoms as well. (#P4)

Participants adopted a range of strategies to mitigate risk including reducing social contact wherever possible, working from home and avoiding public transport.

I'm more of a germaphobe than I was before… [I] wouldn't go out at all and my partner would do the shopping and get the mail and stuff like that because I [have] sort of refused to go anywhere. (#P5)

However, participants also recognized that they had to continue with their work and life, balancing risks and benefits.

I think we've got to be aware… [A]lert, but not alarmed or something like that. (#P4)

Participants took a considered approach to seeking health care. Some participants judged seeking treatment for what they perceived to be minor issues was not worth the potential risk of exposure to COVID‐19.

Why would I want to go and use a health service when it wasn't … nothing was urgent, I wasn't in pain or anything? (#P2)

However, all participants recognized that certain health issues could not be ignored.

But, if I needed to go to the doctor and I was sick, I would go. I think if anything, it's probably quite a safe time to go… because, you know, before this COVID‐19, you'd go into a doctor's waiting room… with people who are nursing colds and flus and you're waiting there in this small room… whereas now we've got social distancing… So, if anything, I think the sort of protocols around visiting a doctor have got better. (#P2)

Also unavoidable were certain face‐to‐face encounters in hospital settings. Some participants reported that they had required hospitalization for unexpected events (eg misadventure or accident) or treatment that could only be carried out in a hospital setting (eg an infusion).

I've actually had some unexpected/eventful health experiences… requiring a trip to ED, surgery and a stay in hospital. (#P2)

When I went in for my infusion they were a little bit more cautious around PPE… Whereas six months ago they didn't use a mask when they were dealing with me… This time they did use a mask every time they engaged with me. (#P7)

On the whole, participants trusted health‐care professionals to advise if a face‐to‐face consultation was necessary and, if so, that COVID‐19 safety procedures would be followed (eg screening questions, social distancing and mask wearing if necessary).

I guess, it's going back to that formal relationship with medical practitioners… So whatever procedures or practices they're adopting in their rooms, you would expect that they're doing that… to the nth degree because it makes perfect sense in a health environment. (#P8)

As part of their usual health management, most participants worked with AHCPs to maintain their mobility and fitness. Following the advent of COVID‐19, they were keen to continue their routine if possible. While some transitioned to online sessions with their exercise physiologists and physiotherapists, other participants with existing relationships trusted AHCPs to take the necessary precautions.

I felt at ease with my physios. And, also, their temperature would get taken each morning, so I knew that [Name] was doing the right thing for his clients, and they were basically spraying and cleaning it down between each patient. (#P6)

### 3.2 Postponing usual care

In response to COVID‐19, some participants postponed certain aspects of their usual care, including visits for mental health care and magnetic resonance imaging (MRI), concerned about the possibility of exposure to COVID‐19 through interactions with other people in a clinical setting.

I waited because I just wasn't comfortable going out in the early stages [of COVID] … to see any health providers during the time… [E]ven, like, mental health wise, I didn't have the mental energy to do anything about it [my mental health]. (#P5)

I, like, didn't [get my MRI] because it seems like a really bad time to go out and get an MRI. (#P1)

Some participants self‐diagnosed and normalized potential emerging symptoms, not actively seeking care unless issues escalated significantly.

[I]t's an MS‐type thing: you don't freak out every time you have a bit of numbness or tingling and thinking that it's a full‐on relapse. It's just something you've just got to ride out and it'll probably go away in due time. (#P2)

It was challenging for participants to decide whether to get treatments such as an infusion of a DMT in a hospital setting where they would have to interact with multiple people.

If my [ocrelizumab] infusion had been set for March or April, I really don't know if I would have [gone] ‐ that would have been a difficult decision. It's one thing to talk about going to a doctor or a neurologist, but taking yourself to a hospital and spending the day in a room with a bunch of other people to get an immunosuppressive drug is a pretty big deal. (#P2)

On the other hand, one participant reflected that the reluctance of some to seek care during the pandemic enabled others to get timely appointments.

I've been stunned that I've had actually less difficulty getting into health‐care providers such as I needed them. (#P4)

### 3.3 New ways of accessing care

Overall, participants were supportive of telehealth consultations during COVID‐19 as they offered a safe and contact‐free option.

I had one session with the neurologist, that was a [video] tele‐consult and that was brilliant. (#P3)

Other reported benefits included better access and greater convenience, especially if transport or fatigue were issues.

It's a lot more convenient just because I live quite a bit out of town to just do it on my phone. (#P5)

If the [medical] problem was causing mobility issues or even extreme fatigue making getting to an appointment difficult then this would certainly be a great option. (#P2)

[T]he neurologist I'm seeing is in Sydney and it was going to be quite difficult to find time to get up there and they offer telehealth [video] and I just went, “Oh, that is fantastic, yeah, let's do that”. (#P4)

However, some negative aspects were raised about consultations via telephone being less personal than a face‐to‐face consultation, and concern about being unable to have a physical examination.

I must say it was better going in to see him because it's a lot more conversational… Although, when I'm on the phone, I can sort of have a list in front of me and work through it. But it's a little bit more natural and ad hoc when you're in with them face‐ to‐face, is what I found. (#7)

I did end up having a neuro appointment by phone and that was OK because he was just giving results from scans, but it would be difficult say if he needed to look at my strength or the way I walk and things like that. It just depends on what you are seeing the doctor for I guess. (#P5)

Video consultations were thought to be better than telephone consultations as they were seen to facilitate better communication through enabling patients to see and read a clinician's body language.

A video meeting where I can see the doctor's face would be significantly better than just the phone. It would help feel more connected and perhaps increase the flow of communication. So much is communicated through body language, facial expressions. (#P7)

Some participants indicated that they saw particular value in having a video consultation for an initial meeting to enable them to determine whether to continue working with the health‐care provider.

For a practitioner I hadn't consulted before I would like a video consult. [You] can learn more from seeing the person in real life and get a better understanding and appreciation of them. (#P8)

In contrast, one participant (#P5) reflected on the importance of having an existing relationship with a clinician and that they would not be comfortable using video for an initial consultation.

I mean its uncomfortable for me to show myself on video to a stranger I guess if I haven't seen them before. (#5)

This participant also described a poor experience with a psychiatric consultation, whereby they could not ‘get eye contact’ because the clinician was not looking at their camera, which left them feeling dissatisfied. They also chose to discontinue their exercise physiology sessions when they were moved online, stating that a ‘person looking at me exercising over video is a bit awkward’.

Participants viewed face‐to‐face consultations as the best environment for discussions about personal health issues, regardless of the type of clinician.

I don't think there's any difference if I'm talking to my GP, neurologist, or any other sort of health professional. I think communication is more complicated than we might think, no matter who the person is, but talking about health issues just magnifies the difficulties because it's often so emotional. (#P4)

Although participants also reported that the health issue of concern also influenced whether they preferred a video or telephone consultation, as some things were simple to address and better suited to a telephone consultation.

I think it is usually better to see people's faces for consultations, especially if I have seen them before… There are differences, of course. GP consults can go by phone easily, especially for prescriptions, certificates, referrals and results and my endocrinologist would probably do phone consults, she usually has me give blood etc a week beforehand. That could work. (#P3)

Most participants felt telehealth was suitable for many, but not all, consultations and would consider incorporating telehealth consultations into their future usual health‐care routine where appropriate.

I think telehealth is a really great initiative…[Implementing] new protocols and new ways of doing things has certainly been very positive…[I] would be really keen to utilise telehealth… [i]n the future, when appropriate, and for different things. (#P2)

## 4 DISCUSSION

Participants in our study attended video and telephone telehealth consultations, and face‐to‐face consultations with a range of health‐care providers including GPs, neurologists, psychiatrists, neuropsychologists, physiotherapists and exercise physiologists. Participants were open to new ways of accessing care and carefully assessed risk when deciding how best to access their health‐care providers. However, they also trusted clinicians to advise when a face‐to‐face consultation was necessary and that they would have appropriate safeguards in place to minimize the potential for infection. This is reflected in the general level of trust Australians have in doctors, nurses and the health system.^29^, ^30^

Participants' experiences of accessing health care during the COVID‐19 pandemic centred on maintaining the usual care and routines that had been established prior to the pandemic to manage a range of symptoms, wherever possible. Decision making was informed by their experiences of living with MS and guided by an assessment of potential risk related to seeking health care. A Canadian study of people with MS' experiences of accessing health care found participants undertook a cost‐benefit analysis exercise to determine whether the energy and resources required to seek care were ‘worth it’, especially as fatigue was a common symptom. This meant that some participants practised health‐care avoidance until they experienced a health crisis.^15^ While recognizing that people with MS are experts in self‐management, symptom normalization can impact on decision making^15^ and lead to inappropriate postponement of care. Concerns about exposure to COVID‐19 may further encourage postponement. Continuation of usual care is essential for people with MS as they utilize multiple health‐care professionals to manage their chronic symptoms, stay active and maintain their independence.^17^, ^18^, ^31^ Addressing psychosocial issues during stressful times is particularly important and relational continuity in health care is highly valued by people with MS as a source of on‐going psychological support, given that MS is a fluctuating condition.^18^, ^32^

The COVID‐19 pandemic has brought about a rapid increase in telehealth use in Australia and globally.^33^ Prior to the pandemic, telehealth had been shown to be useful in the management of neurological conditions,^34^ including MS.^35^ The key benefits and limitations of telehealth identified by participants in our study align broadly with those in the literature and included safety, convenience and access, tempered by the inability to have a physical examination and difficulty communicating when unable to read a clinician's body language. An MS health service in the UK that rapidly transitioned to online care in response to the pandemic reported both people with MS and clinicians found video consultations more effective than telephone consultations, citing eye contact and the opportunity to pick up subtle facial communication cues as important. They successfully put in place tools for patients to self‐assess their neurological function and quality of life prior to a video consultation. The tools included a web‐based Expanded Disability Status Scale (EDSS), a self‐administered timed 25‐foot walk, a 9‐hole peg test and the MSIS‐29 quality of life survey.^36^

In contrast, a recent survey in Italy looking at teleconsultations for people with MS during the COVID‐19 pandemic^37^ found that, while they appreciated their role during the pandemic, participants had a strong preference for face‐to‐face evaluations, citing the need for human empathy with their neurologist and belief that a physical examination would lead to better clinical outcomes. Most stated they would not consider alternating telehealth and face‐to‐face visits in the future. In our study, participants felt that while teleconsultation could not replace face‐to‐face consultation, particularly when physical examination is needed, it offered convenience and greater access to health‐care professionals, thus enabling a safe and contact‐free option. Participants were comfortable with the idea of incorporating telehealth, where appropriate, into their future health care.

Teleconsultation is thought to be best‐suited to consultations between patients and clinicians who have an established therapeutic relationship, and this is mirrored in the current telehealth eligibility criteria in place in relation to general practice consultations in Australia.^11^ However, all participants in our study (except one) felt they could be useful for initial consultations as well.

Most participants found that while telephone consultations were efficient, they were less personal as patients were unable to read their clinicians' body language, making it more difficult to understand meaning. However, one participant found they were able to focus more clearly and refer to their own notes on the telephone. Most participants in our study preferred video consultation, consistent with previous studies that have found higher levels of satisfaction among patients and staff using video links.^38^, ^39^ A remote consultation can change the interaction between patient and clinician and may adversely affect the flow of conversation,^40^ especially if there is no body language to contextualize the situation.^41^ However, there is also considerable evidence that telephone consultations are effective and require less technology, meaning that video options should supplement, rather than replace, them.^38^ While not raised as an issue in our research, social inequities may be accentuated with telehealth through disparities in access to the Internet and the capacity to use this modality,^42^, ^43^, ^44^ especially for people with a disability^45^ and the elderly,^46^ highlighting the need for skills training and addressing access issues.

Clinicians and patients familiar with video consultation may experience difficulties with flow of conversation but develop strategies over time to overcome this.^40^ Both patients and clinicians new to telephone consultation can find the absence of non‐verbal communication challenging and have reported difficulties in clarifying patient complaints.^41^ Our study was undertaken in the early days of telehealth, when it would be expected that participants and clinicians had not yet become skilled in the more precise and clear visualizing language needed to communicate in remote consultations.

Reflecting on the rapid rollout of telehealth across Australia in response to the COVID‐19 pandemic, there is significant uncertainty about how telehealth consultations will fit into standard clinical care in the future. Workflows have been quickly reorganized during the COVID‐19 crisis and there is a pressing need for further evaluation, support and guidance.^38^, ^40^ Knowledge and capacity gaps have been exposed within the current health workforce highlighting the need for specific training and support for remote consultation skills.^33^

### 4.1 Strengths and limitations

Patient and public involvement in research has been found to increase the quality and relevance of research,^47^ as well as enhancing a study's credibility and legitimacy.^48^ Strengths of this study include its focus on the perspectives and experiences of people with MS accessing health care through the COVID‐19 pandemic and the involvement of people with MS as research team members. We found that having a peer with MS as an interviewer assisted with building rapport with participants although it might be argued that participants provided biased socially desirable answers. We chose not to include a validated measure of disability in our description of participants, respecting the recommendation of our team members with MS. While measures such as the Expanded Disability Status Scale (EDSS) and Multiple Sclerosis Functional Composite (MSFC) are widely used to measure functional end points for people with MS,^49^ the use of these to measure disease progression is a source of controversy in the MS community.^50^, ^51^ For example, the EDSS focuses on lower body mobility, while neglecting to account for upper body mobility or disability related to cognition.

While our study included people with MS manifesting in a variety of ways, care must also be taken with regard to the transferability of this study due to the sample being drawn from one geographical location and the inclusion of only those who spoke English. We reached data saturation and tested this through conducting an additional two interviews, with a final sample size of eight. A larger sample might be required to achieve saturation in a larger geographic area. While the study does inform thinking about the broader rollout of telehealth in Australia, a limitation is that it was conducted in the early months of telehealth when a novice effect may have been operating. Further research in this area, including with people for whom English is their second language, is needed.

### 4.2 Conclusion

Personal risk assessment and trust in health‐care professionals are determinants of the mode through which people with MS accessed health care during the COVID‐19 pandemic. As an adjunct to face‐to‐face consultations telehealth undoubtedly has a place in providing usual care for people with MS. The uptake of telehealth in Australia has been rapid, offering patients and clinicians protection from disease transmission; and for people with MS, convenience and access to health‐care professionals—providing a safe, contact‐free option during the pandemic. Telehealth has a role in any comprehensive treatment plan, especially in a geographically dispersed country such as Australia. People with MS may find specific value in video consultations, which enable visualization of physical function. While technology can facilitate access for some people with MS, addressing the digital divide to ensure access for all is important. There is a need for training and support for all clinicians to conduct remote consultations, including learning how to use visual language in their communication with patients.

## CONFLICT OF INTEREST

The authors declare no potential conflicts of interest with respect to the research, authorship and/or publication of this article.

## AUTHORS' CONTRIBUTIONS

JD, JDr and AP designed the study. Following discussion with all authors, it was refined. JDr and AP completed data collection. Initial data analysis was undertaken by JDr and AP; it was discussed and further refined with JD and SH. It was then discussed with the whole team and further refined. AP and JDr led the drafting of the manuscript. JD, CP and CL made significant contribution to drafting and revision of the manuscript. SH, VF, KC and ME contributed to revisions and reflection on relevance to people with MS.

## ETHICAL APPROVAL

This study was approved by the Australian National University Human Research Ethics Committee (2020/237).

## Data Availability

The data are not publicly available due to privacy and ethical restrictions.
